# Recent Advances in the Roles of Autophagy and Autophagy Proteins in Host Cells During *Toxoplasma gondii* Infection and Potential Therapeutic Implications

**DOI:** 10.3389/fcell.2021.673813

**Published:** 2021-06-09

**Authors:** Carlos S. Subauste

**Affiliations:** ^1^Division of Infectious Diseases and HIV Medicine, Department of Medicine, Case Western Reserve University, Cleveland, OH, United States; ^2^Department of Pathology, Case Western Reserve University, Cleveland, OH, United States

**Keywords:** cell signaling, CD40, IFN-γ, macrophage, endothelial, epithelial

## Abstract

*Toxoplasma gondii* is an obligate intracellular protozoan that can cause encephalitis and retinitis in humans. The success of *T. gondii* as a pathogen depends in part on its ability to form an intracellular niche (parasitophorous vacuole) that allows protection from lysosomal degradation and parasite replication. The parasitophorous vacuole can be targeted by autophagy or by autophagosome-independent processes triggered by autophagy proteins. However, *T. gondii* has developed many strategies to preserve the integrity of the parasitophorous vacuole. Here, we review the interaction between *T. gondii*, autophagy, and autophagy proteins and expand on recent advances in the field, including the importance of autophagy in the regulation of invasion of the brain and retina by the parasite. We discuss studies that have begun to explore the potential therapeutic applications of the knowledge gained thus far.

## Introduction

Intracellular pathogens have developed a broad range of strategies to survive within host cells. *Toxoplasma gondii* is an important example of these pathogens. *T. gondii* secretes proteins during its interaction with host cells that: (i) Enable the formation of a parasitophorous vacuole (PV) that avoids the classical pathway of phagolysosomal fusion; (ii) Activate host cell signaling cascades that negatively regulate autophagic targeting of the PV. As a result, the parasite avoids lysosomal degradation mediated by constitutive autophagy and prevents its complete eradication when autophagy is stimulated by CD40; (iii) Impair the ability of interferon-gamma (IFN-γ) to activate autophagosome-independent effector mechanisms directed against the PV. We provide a brief overview of early work on the role of macroautophagy (referred herein as autophagy) and autophagy proteins in *T. gondii* infection and discuss in more detail recent discoveries that include the role of autophagy in the regulation of invasion of the brain and retina by *T. gondii*, identification of a signaling pathway that causes sustained blockade of autophagic targeting of the parasite, identification of a molecular link between IFN-γ and autophagy proteins (ATG) in human cells as well as the role of ATG8 orthologs in the cell-autonomous control of *T. gondii* induced by IFN-γ. We also discuss the potential implications for new therapeutic approaches against toxoplasmosis.

## *Toxoplasma gondii* Infection

*Toxoplasma gondii* is an apicomplexan protozoan of worldwide distribution. *T. gondii* is believed to remain in the infected host for life. One-third of the world population is chronically infected with *T. gondii* (Montoya and Liesenfeld, 2004). Although the infection is usually asymptomatic, *T. gondii* can cause ocular toxoplasmosis in immunocompetent and immunosuppressed patients and encephalitis in immunocompromised individuals (Montoya and Liesenfeld, 2004). In addition, pregnant women acutely infected with *T. gondii* can transmit the infection to the unborn baby (Montoya and Liesenfeld, 2004). Congenital toxoplasmosis can result in severe ocular and neurologic sequelae as well as abortion (Montoya and Liesenfeld, 2004). Most of the strains of *T. gondii* isolated in North America and Europe fall into three clonal lineages: I, II, and III. Atypical strains that can have a higher propensity to cause disease have been reported in South America (Gilbert et al., 2008).

There are various forms of the parasite during its life cycle that include: (i) the tachyzoite that infects almost any nucleated cell of the host; (ii) the tissue cyst (containing bradyzoites) that persists in tissues of infected hosts during the chronic phase of the infection; and (iii) the oocyst (containing sporozoites) that is produced in the intestine of felines (the definitive host) during the sexual cycle of the parasite. Humans and other intermediary hosts become infected by ingesting tissue cysts or oocysts. This is followed by the release of bradyzoites or sporozoites into the intestinal lumen, invasion of intestinal epithelial cells, transformation into tachyzoites, and dissemination *via* the blood and lymphatic.

Tachyzoites of *T. gondii* invade mammalian cells and form a PV within these cells. This process is dependent on the secretion of contents from parasite organelles: (i) Micronemes that secrete micronemal proteins (MICs), adhesins that bind host cell membrane receptors (Carruthers and Tomley, 2008). (ii) Rhoptries that secrete rhoptry neck proteins that form the moving junction (Bradley and Sibley, 2007). This structure anchors the parasite to the host cell cytoskeleton and excludes host cell type I transmembrane proteins from the membrane surrounding the parasite (Mordue et al., 1999). The exclusion of these proteins could explain the first strategy identified for parasite survival: avoiding the classical pathway of phagolysosomal fusion (Mordue et al., 1999). Rhoptries also inject rhoptry kinases (ROPs) into the host cell cytoplasm that manipulate host cell signaling (Hakimi et al., 2017). (iii) Dense granules that release GRA proteins that maintain the intravacuolar network, anchor to the PV membrane (PVM), and interact with the host cytosol (Cesbron-Delauw et al., 2008; Hakimi et al., 2017). As explained later, proteins from these organelles are also instrumental in setting in motion signaling cascades that subvert cell autonomous mechanisms of parasite eradication.

## CD40 and Autophagy-Dependent Killing of *T. gondii*: Relevance to the Regulation of Invasion of the Brain and Retina by the Parasite

Studies in humans and mice revealed that CD40 and its main ligand CD154 are central for protection against cerebral and ocular toxoplasmosis (Subauste et al., 1999; Reichmann et al., 2000; Portillo et al., 2010). Although studies in patients with congenital deficiency of CD154 (X-linked hyper immunoglobulin M syndrome) linked defective T cell priming and type 1 cytokine production to susceptibility to toxoplasmosis (Subauste et al., 1999), work done in CD40^–/–^ and CD154^–/–^ mice showed that these animals had increased susceptibility to cerebral and ocular toxoplasmosis despite seemingly unimpaired expression of IFN-γ (Reichmann et al., 2000; Portillo et al., 2010). The likely mechanisms of protection against cerebral and ocular toxoplasmosis identified in mice are as follows: (i) CD40-induced toxoplasmacidal activity in macrophages/microglia (Reichmann et al., 2000; Portillo et al., 2010); (ii) recently identified induction of toxoplasmacidal activity in neural endothelial cells accompanied by reduced invasion of the brain and retina by the parasite (Portillo et al., 2019); (iii) reduced anti-*T. gondii* antibody production (Portillo et al., 2021); (iv) protection against CD8^+^ T cell exhaustion (Bhadra et al., 2011). CD8^+^ T cells, important mediators of protection against *T. gondii*, undergo exhaustion (loss of functional capacities) during the chronic phase of *T. gondii* infection, a process that is mediated by program death-1 (Bhadra et al., 2011). Blockade of program death-1 rescues CD8^+^ T cells, an effect that is dependent on the CD40-CD154 pathway (Bhadra et al., 2011). However, it is important to note that cerebral and ocular toxoplasmosis in CD40^–/–^ and CD154^–/–^ mice precede the development of CD8^+^ T cell exhaustion.

**FIGURE 1:**
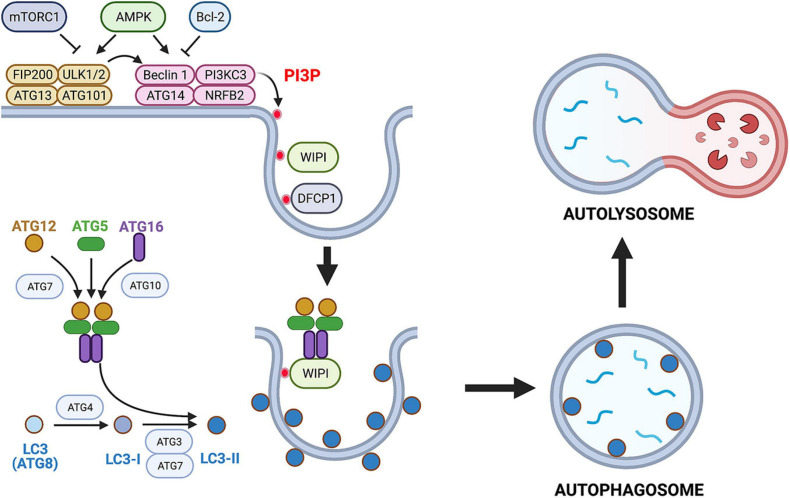
Overview of the autophagy pathway. Autophagy is a constitutive and well-conserved process of lysosomal degradation. Autophagy is triggered by activation of ULK1 that forms a complex with ATG13, ATG101, and FIP200. ULK1 is under control of AMPK and mechanistic target of rapamycin complex 1 (mTORC1). A decrease in energy status activates ULK1 *via* AMPK leading to autophagy stimulation, whereas nutrient-rich conditions inhibit ULK1 *via* mTORC1. Membrane-associated ULK1 complex recruits and activates the PI3KC3 complex that also contains Beclin 1, ATG14, nuclear receptor binding factor 2 (NRBF2), as well as p150 and activating molecule in BECN1-regulated autophagy protein 1 (AMBRA1) (not shown). Bcl-2 inhibits pro-autophagic effect of Beclin 1. Activation of PI3KC3 complex causes local production of PI3P that recruits PI3P-binding proteins double FYVE domain-containing protein 1 (DFCP1) and WD-repeat protein interacting with phosphoinositides (WIPI). These scaffold proteins mediate membrane remodeling. Active PI3KC3 complex and WIPI promote expansion of isolation membrane through recruitment of ATG proteins. These proteins function as two ubiquitin-like conjugation systems: (i) ATG7 and ATG10 that conjugate ATG5 to ATG12 and ATG16. (ii) ATG3 and ATG7 together with ATG12–ATG5–ATG16 complex induce lipidation (phosphatidylethanolamine conjugation) of LC3 (ATG8). Lipidated LC3 (LC3-II) binds to several autophagy receptors promoting substrate uptake. Sequestration of the cargo by autophagosomes is followed by fusion with lysosomes resulting in formation of an autolysosome and lysosomal degradation of the cargo. This figure is a simplified overview of some of central components of autophagy pathway in mammalian cells. Figure was generated using BioRender.com.

CD40 kills a type I strain of *T. gondii* in human and mouse macrophages, a process independent of IFN-γ and effector responses downstream of this cytokine, but is instead caused by autophagy-mediated degradation of the parasite (Andrade et al., 2006). The molecular events involved in autophagy, including the role of this process in *T. gondii* infection, have been reviewed elsewhere (Mizushima et al., 2010; Besteiro, 2019; Subauste, 2019; Melia et al., 2020; Nakatogawa, 2020). A schematic summary of autophagy is shown in Figure 1.

**FIGURE 2:**
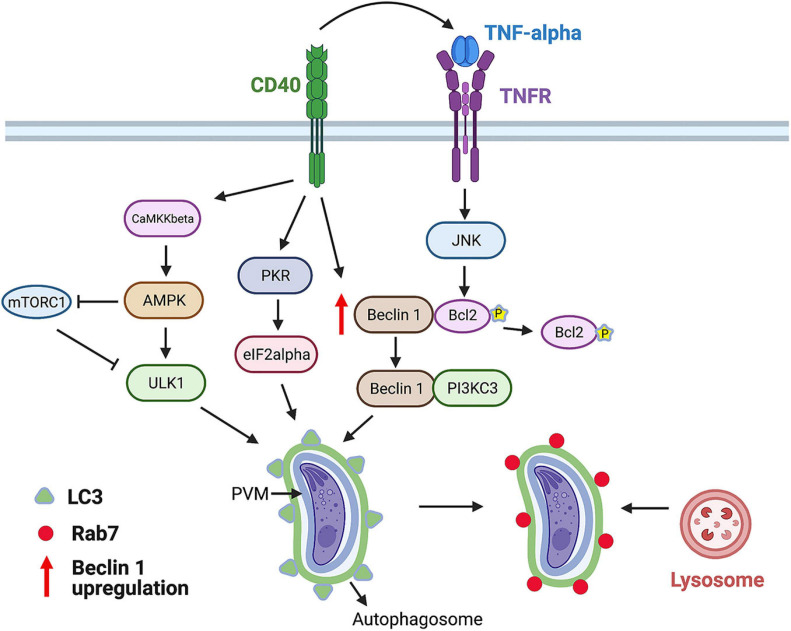
CD40 induces killing of *T. gondii* via autophagy. CD40 ligation stimulates autophagy by activating ULK1, upstream kinase in the autophagy pathway. This process is dependent on calcium sensor CaMKKβ that activates energy/nutrient sensor AMPK. Besides activating ULK1, AMPK inhibits mTORC1, an inhibitor of autophagy. In addition, CD40 stimulates autophagy by triggering activation of pro-autophagy proteins PKR and eIF2α, upregulating autophagy protein Beclin 1, and releasing Beclin 1 from its negative regulator Bcl-2 after Bcl-2 phosphorylation induced by TNF receptor (TNFR)-JNK signaling. CD40 induces recruitment of LC3 around parasitophorous vacuole membrane (PVM) followed by Rab7-dependent fusion with lysosomes and killing of parasite. This process requires ULK1, Beclin 1, PI3KC3, ATG5, ATG7, and lysosomal enzymes. Figure was generated using BioRender.com.

CD40 stimulates autophagy through events that include: (i) Activation of Unc-51-like kinase 1 (ULK1), the upstream kinase that stimulates autophagy, through a process dependent on calcium/calmodulin-dependent kinase kinase-β (a sensor of intracellular Ca^2+^) and AMP-activated protein kinase (an energy and nutrient sensor) (Figure 2); (ii) dissociation of the autophagy protein Beclin 1 (ATG6) from its negative regulator Bcl-2; (iii) upregulation of Beclin 1; and (iv) activation of protein kinase double-stranded RNA-dependent (PKR) and the α subunit of eukaryotic initiation factor 2 α, proteins that stimulate autophagy (Ogolla et al., 2013; Liu et al., 2016; Figure 2). As a result, the autophagy protein LC3 (ATG8) is recruited around the PV, a process that is followed by vacuole–lysosomal fusion (VLF) as assessed by an accumulation of the late endosomal and lysosomal markers Rab7, mannose-6-phosphate receptor, CD63, lysosomal-associated membrane protein 1 (LAMP-1), and cathepsin D (Andrade et al., 2006). Parasite killing is dependent on ULK1, Beclin 1, and its interacting partner phosphatidylinositol 3-kinase catalytic subunit type 3 (PI3KC3), the autophagy proteins ATG5 and ATG7, and requires the activity of lysosomal enzymes, indicating that CD40 kills *T. gondii* in macrophages *via* autophagy (Andrade et al., 2006; Portillo et al., 2010; Liu et al., 2016). These findings have *in vivo* relevance because Beclin 1-deficient mice (*BECN1*^±^), mice with deficiency of ATG7 in myeloid cells (Atg7^flox/flox^-Lyz-M Cre mice) and mice deficient in PKR (PKR^–/–^), exhibited increased susceptibility to cerebral and ocular toxoplasmosis (Portillo et al., 2010; Ogolla et al., 2013).

CD40 induces killing of type I and II strains of *T. gondii* in various human and mouse non-hematopoietic cells, including endothelial cells, through a mechanism dependent on the same components of the autophagy pathway identified in macrophages (Van Grol et al., 2013). As described later, consistent with the evidence that endothelial cells are the portal of entry into the central nervous system (CNS), CD40-driven autophagic killing in endothelial cells has been recently linked to regulation of invasion of the brain and eye, the two main organs affected by toxoplasmosis (Portillo et al., 2019).

Regardless of the route of infection, *T. gondii* circulates in the blood within infected leukocytes (including CD11b^+^ monocytes and dendritic cells) and as extracellular tachyzoites, a process that enables the parasite to invade the CNS (Courret et al., 2006; Lambert et al., 2006; Konradt et al., 2016). Neural endothelial cells become infected, and parasite replication within these cells followed by parasite egress into the neural parenchyma is likely the main mechanism of invasion of the CNS (Konradt et al., 2016). CD40^–/–^ mice exhibited lower parasite load in the brain and retina since the initial stages of infection with *T. gondii*, suggesting that CD40 restricts invasion of these organs (Portillo et al., 2019). CD40^–/–^ transgenic mice where CD40 expression is rescued in endothelial cells were used to examine further the role of endothelial CD40 during the development of cerebral and ocular toxoplasmosis. The presence of CD40 in these cells resulted in diminished parasite load and histopathology in the brain and retina after *T. gondii* infection (Portillo et al., 2019). Moreover, a lower parasite load was also detected after intravenous injection of *T. gondii*-infected CD11b^+^ monocytes or dendritic cells, indicating that endothelial cell CD40 reduced parasite invasion of the brain and retina (Portillo et al., 2019). The protective effect of endothelial CD40 was dependent on the presence of circulating infected leukocytes because no reduction in parasite load in the brain and retina was noted after challenge with extracellular tachyzoites (Portillo et al., 2019). Rather than reducing transmigration of infecting leukocytes across endothelial cells, CD40 likely conferred protection against cerebral and ocular toxoplasmosis because the presence of CD40 reduced the number of parasite foci in neural endothelial cells, diminishing invasion of the CNS (Portillo et al., 2019). *In vitro* studies revealed that endothelial cells acquired toxoplasmacidal activity upon interaction with *T. gondii-*infected dendritic cells or macrophages (Portillo et al., 2019). This effect was dependent on the expression of CD40, ULK1, and Beclin 1 in endothelial cells and led to the recruitment of LC3 and LAMP-1 around the PV of infected endothelial cells. During the interaction between infected leukocytes and endothelial cells, CD154 did not seem to be the major trigger for the autophagic killing of *T. gondii* in CD40^+^ endothelial cells (Portillo et al., 2019). Rather, the expression on infected leukocytes of inducible heat shock protein 70, a protein reported to function as a ligand for CD40 (Becker et al., 2002; Shevtsov et al., 2014), seemed to induce toxoplasmacidal activity in endothelial cells (Portillo et al., 2019). Finally, the reduction in parasite invasion of the brain and retina was dependent not only on the expression of CD40 by endothelial cells but also on the expression of Beclin 1 and the expression of inducible heat shock protein 70 in dendritic cells (Portillo et al., 2019). These results indicate that CD40 and autophagy enhance protection against cerebral and ocular toxoplasmosis also by restricting parasite invasion of neural tissue.

Immune mechanisms of protection against pathogens, including *T. gondii*, do not fully overlap in humans and mice. For example, protection against *T. gondii* in mice requires IFN-γ and its downstream molecule signal transducer and activator of transcription 1 (STAT1) (Gavrielescu et al., 2004; Lieberman et al., 2004). A markedly different phenotype occurs in patients with an autosomal dominant defect in IFN-γR1 that results in deletion of the STAT1 binding site (Janssen et al., 2002). These patients are not susceptible to toxoplasmosis, although their macrophages are unable to display anti-*T. gondii* activity in response to IFN-γ (Janssen et al., 2002). Incubation with high concentrations of tumor necrosis factor-alpha (TNF-α) partially restored antimicrobial activity of macrophages from these patients, suggesting a protective role for TNF-α in these patients (Janssen et al., 2002). CD40 may contribute to IFN-γ-independent mechanisms of protection in humans (Subauste, 2009), as CD40 induces killing of *T. gondii* independently of IFN-γ, STAT1, and effector molecules downstream of IFN-γ [immunity-related GTPases (IRGs); nitric oxide synthase 2] and CD40 induces TNF-α production (Andrade et al., 2005; Subauste and Wessendarp, 2006).

## *Toxoplasma gondii* Manipulates Host Cell Signaling to Avoid Autophagic Targeting: Mechanism of Persistent Blockade of Autophagic Degradation and Relevance to Invasion of the Brain and Retina

**FIGURE 3:**
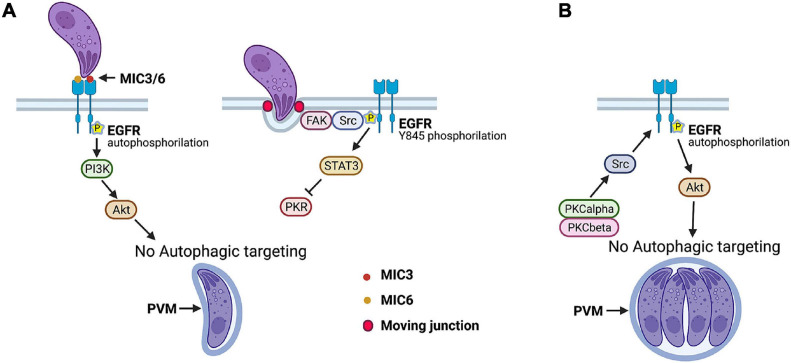
*Toxoplasma gondii* manipulates host cell signaling cascades that prevent autophagic targeting of parasite. **(A)** Parasite adhesins MIC3 and MIC6 induce EGFR autophosphorylation that triggers PI3K-dependent activation of Akt, a negative regulator of autophagy. FAK, a cytoplasmic non-receptor tyrosine kinase, is activated during invasion of host cell at level of moving junction. This causes Src-dependent transactivation of EGFR (Y845 phosphorylation) and STAT3 signaling that prevents activation of pro-autophagy proteins PKR and eIF2α. **(B)** *T. gondii* maintains a blockade of autophagic targeting during its intracellular state by activating serine/threonine kinases PKCα/PKCβ that in turn maintain Src signaling, EGFR autophosphorylation, and Akt activation. Blockade of any of signaling molecules activated by *T. gondii* results in autophagic targeting of PV and killing of parasite. This process is dependent on ULK-1, Beclin 1, ATG7, and lysosomal enzymes. Figure was generated using BioRender.com.

Under basal conditions, *T. gondii*-infected cells accumulate LC3^+^ structures around the PV without leading to autophagic targeting of the parasite (Wang et al., 2009), indicating that the parasite avoids targeting by autophagy. Indeed, *T. gondii* activates epidermal growth factor receptor (EGFR) and Src signaling during the invasion of an infected host cell to prevent its degradation by autophagy. MIC3 and MIC6, parasite proteins that contain EGF-like domains, cause rapid autophosphorylation of EGFR in human and mouse host cells followed by activation of phosphoinositide 3-kinase (PI3K), production of phosphatidylinositol 3,4,5 triphosphate around the PV, and activation of Akt, a negative regulator of autophagy (Muniz-Feliciano et al., 2013; Figure 3A). Blockade of any of the components of this cascade caused accumulation of LC3 and LAMP-1 around the PV, entrapment of the PV by a double membrane structure with characteristics compatible with those of an autophagosome, and killing of type I and II strains of *T. gondii* in human and rodent hematopoietic and non-hematopoietic cells that were dependent on the autophagy proteins ULK1, Beclin 1, ATG7, and lysosomal enzymes (Muniz-Feliciano et al., 2013).

The formation of the moving junction triggers a second signaling cascade that prevents autophagic targeting of *T. gondii*: activation of the cytoplasmic non-receptor tyrosine kinase focal adhesion kinase and its interacting partner Src that in turn causes ligand-independent transactivation of EGFR (Y845 phosphorylation of EGFR) (Portillo et al., 2017; Figure 3A). This pathway is responsible for the early phase of activation of STAT3, a negative regulator of autophagy (Portillo et al., 2017; Figure 3A). STAT3 acts by blocking the activation of the pro-autophagy protein PKR and its downstream signaling molecule eIF2α (Portillo et al., 2017). Inhibition of the focal adhesion kinase–Src–pY845 EGFR–STAT3 pathway results in the autophagic killing of type I, type II, and atypical strains of *T. gondii* in hematopoietic and non-hematopoietic human and rodent cells (Portillo et al., 2017).

Studies in transgenic mice that express a dominant-negative (DN) mutant of EGFR that impairs EGFR autophosphorylation and EGFR transactivation revealed the importance of manipulating host cell signaling in the development of cerebral and ocular toxoplasmosis. Expression of DN EGFR restricted to endothelial cells was accompanied by a reduction in parasite load and histopathology in the brain and retina after *T. gondii* infection (Lopez Corcino et al., 2019b). A lower parasite load in the CNS was also noted after intravenous challenge with infected dendritic cells or extracellular tachyzoites. The presence of DN EGFR reduced the number of foci of infection in neural endothelial cells (Lopez Corcino et al., 2019b). Moreover, DN EGFR in these cells led to spontaneous recruitment of LC3 around *T. gondii*, VLF, and parasite killing dependent on ULK1 and lysosomal enzymes (Lopez Corcino et al., 2019b). Autophagy likely explains the protective role of DN EGFR, as *in vivo* administration of the autophagy inhibitor 3-methyl adenine prevented DN EGFR mice from exhibiting reduced CNS invasion (Lopez Corcino et al., 2019b). Thus, EGFR enhances *T. gondii* invasion of the CNS likely by promoting parasite survival in endothelial cells through avoidance of autophagic targeting. Moreover, these studies together with those on mice that express CD40 restricted to endothelial cells support the central role of autophagy as a process that regulates *T. gondii* invasion of the CNS.

The survival strategies described earlier are operative during the invasion of host cells by *T. gondii*. However, autophagy is a constitutive process, indicating that the parasite likely uses additional mechanisms to maintain a blockade of autophagic targeting. Indeed, *T. gondii* causes prolonged EGFR autophosphorylation in mammalian cells that is functionally relevant, as the addition of EGFR tyrosine kinase inhibitors (TKIs) 6 h after challenge with *T. gondii* resulted in the killing of the parasite (Lopez Corcino et al., 2019a). This process involved entrapment of the PV by a double-membrane structure compatible with an autophagosome, accumulation of LC3 and LAMP-1 around the PV, and pathogen killing dependent on ULK1, Beclin 1, and lysosomal enzymes (Lopez Corcino et al., 2019a). *T. gondii* induces prolonged EGFR autophosphorylation and activation of its downstream molecule Akt through a cascade that consists of the cytosolic serine/threonine kinases protein kinase C α (PKCα) and PKCβ that cooperate to sustain Src activation that drives prolonged EGFR autophosphorylation (Lopez Corcino et al., 2019a; Figure 3B). All these signaling molecules promote parasite survival, as not only inhibition of EGFR but also of PKCα, PKCβ, Src, and Akt in cells previously infected with *T. gondii* led to parasite killing (Lopez Corcino et al., 2019a). Thus, whereas *T. gondii* MIC3 and MIC6 act as an early switch for EGFR autophosphorylation, PKCα/β–Src signaling maintains EGFR autophosphorylation, ensuring the non-fusogenic nature of the PV and parasite survival.

## Interferon-Gamma Restricts *T. gondii* Cells Through Autophagy-Independent Effects of Autophagy Proteins: Role of ATG8 Orthologs, Ubiquitin, and the Molecular Link Between IFN-γ and the ATG Pathway

### Mouse Cells

**FIGURE 4:**
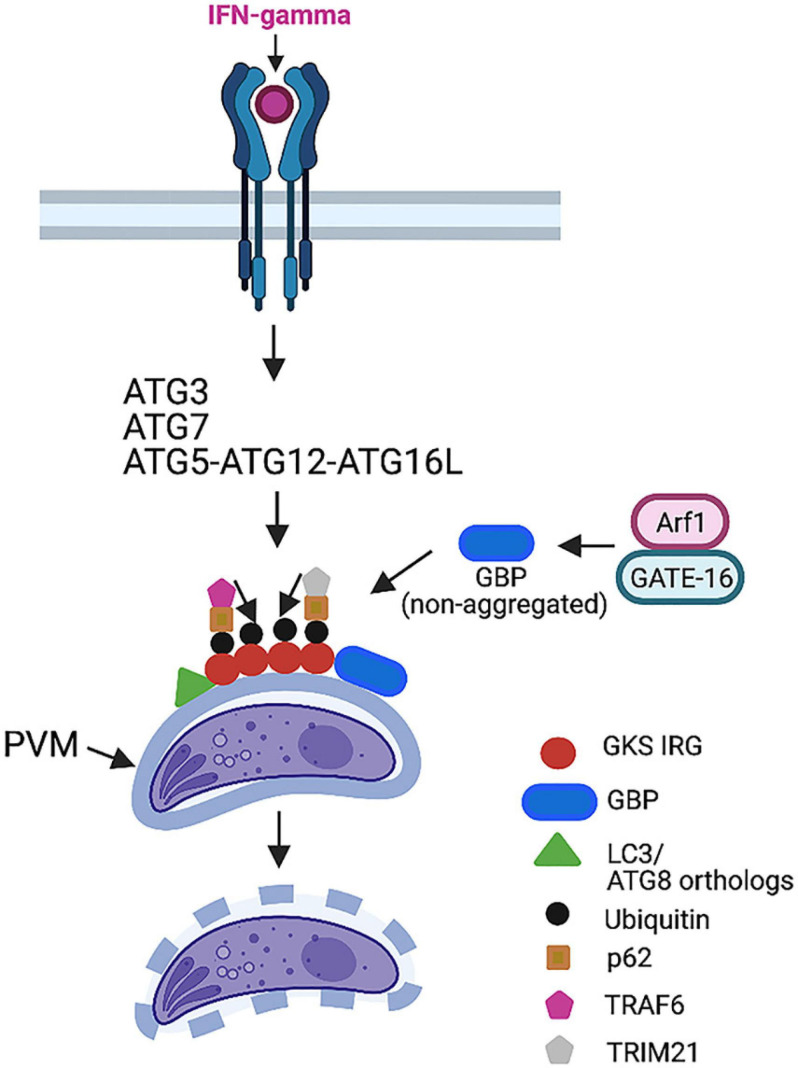
IFN-γ-induces killing of type II and III strains of *T. gondii* in mouse cells. IFN-γ triggers recruitment of GKS IRGs to parasitophorous vacuole membrane (PVM), a process that requires ATG3, ATG7, and ATG12–ATG5–ATG16L. LC3 and ATG8 orthologs are recruited to PVM. IRGs promote deposition of ubiquitin, p62, and the E3 ubiquitin ligase TRAF6. p62 and TRAF6 further promote sustained decoration of PVM with ubiquitin. This process leads to delivery of GBPs to PVM. The end result is disruption of PVM and death of parasite. TRIM21 is another E3 ubiquitin ligase recruited by p62 that promotes ubiquitination. However, it does not seem to drive vesiculation of PVM. GATE-16 activates Arf1, enabling GBP to remain in a non-aggregated form, thus promoting GBP loading onto PVM. This process does not occur in cells infected with type I strains of *T. gondii* because ROP5/ROP18/GRA7 inactivate IRGs preventing their loading onto PVM. Figure was generated using BioRender.com.

IFN-γ activates mechanisms that target the PV triggering cell-autonomous control of the parasite. In the case of mouse macrophages and fibroblasts (but not in human cells), certain autophagy proteins (ATG3, ATG5, ATG7, ATG12, and ATG16L) are required for recruitment to the PVM of effector GKS subfamily of IRGs (Zhao et al., 2008; Khaminets et al., 2010; Choi et al., 2014; Ohshima et al., 2014; Figure 4). This process induces deposition of ubiquitin and p62 on the PVM, followed by p62-dependent recruitment of guanylate-binding proteins (GBPs) (Haldar et al., 2015; Figure 4). The end result is vesiculation and rupture of the PVM and death of susceptible strains (types II and III) of *T. gondii* (Martens et al., 2005; Ling et al., 2006; Khaminets et al., 2010; Haldar et al., 2015). Type I strains avoid this process because ROP18, ROP5, ROP17, and GRA7 from these virulent strains prevent IRG recruitment to the PVM (Fentress et al., 2010; Fleckenstein et al., 2012; Etheridge et al., 2014; Reese et al., 2014). Disruption of the PVM in cells infected with type II and III strains of *T. gondii* does not represent *bona fide* autophagy, as it is not dependent on ULK1, ATG9, and ATG14L (Choi et al., 2014) and is not mediated by lysosomal degradation (Andrade et al., 2006; Van Grol et al., 2013; Choi et al., 2014).

Ubiquitin is a key component of the machinery that mediates the killing of *T. gondii* in IFN-γ-activated cells. Deposition of ubiquitin in the PVM was shown to be mediated not only by IRGs but also by p62 and the E3 ubiquitin ligase TNF receptor-associated factor 6 (TRAF6) (Haldar et al., 2015). Ubiquitin binds p62 that, in turn, recruits TRAF6 (Haldar et al., 2015; Figure 4). Both p62 and TRAF6 establish a positive feedback loop that contributes to the rapid and sustained decoration of the PV with ubiquitin, resulting in the delivery of GBPs to the PV and parasite killing (Haldar et al., 2015). The tripartite motif protein 21 (TRIM21) is another E3 ubiquitin ligase that was recruited to the PV and promoted ubiquitin deposition in IFN-γ-treated cells (Haldar et al., 2015; Foltz et al., 2017; Figure 4). Whereas TRIM21 localized to GBP1-positive PV, TRIM21 did not promote vesiculation of the PVM (Foltz et al., 2017). However, TRIM21 was still able to restrict parasite replication (Foltz et al., 2017). In addition, studies in TRIM21^–/–^ mice revealed that TRIM21 played an *in vivo* role in protection against *T. gondii* (Foltz et al., 2017). It is possible that the *in vivo* protective effect of this molecule is also mediated by the modulation of cytokine production (Foltz et al., 2017).

Mammalian cells express various ATG8 orthologs, including LC3A, LC3B, LC3C, gamma-aminobutyric (GABA)-A-receptor-associated protein (GABARAP), GABARAP-like1 (GABARAPL1), and GABARAPL2 (GATE-16). However, the PV is heavily decorated with LC3 in IFN-γ-activated mammalian cells; studies in mouse fibroblasts that lacked LC3A and LC3B revealed that LC3 proteins were dispensable for the control of *T. gondii* induced by IFN-γ (Sasai et al., 2017). In contrast, mouse fibroblasts that lacked GATE-16, GABARAP, and GABARAPL1 showed a marked defect in the recruitment of Irga6, ubiquitin, p62, and GBP1-5 to the PV as well as impaired parasite clearance after stimulation with IFN-γ (Sasai et al., 2017). The recruitment of GBP was dependent on the small GTPase ADP ribosylation factor 1 (Arf1) (Sasai et al., 2017). GATE-16 associated with Arf1 promoted its activation and, in turn, the recruitment of GBP (Sasai et al., 2017; Figure 4). Similar to mice with deficiency in ATG5, ATG7, or ATG16L targeted to phagocytes (Zhao et al., 2008; Choi et al., 2014), GATE-16-deficient mice died during the acute phase of *T. gondii* infection (Sasai et al., 2017). Thus, GATE-16 is central for resistance against *T. gondii* infection.

### Human Cells

**FIGURE 5:**
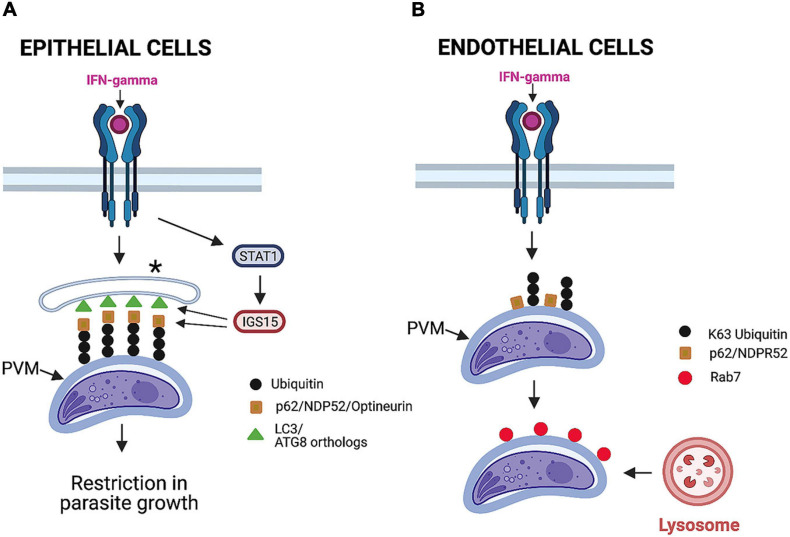
Mechanisms induced by IFN-γ to restrict *T. gondii* in human cells. **(A)** In epithelial cells, IFN-γ promotes deposition of ubiquitin, adaptor proteins (p62, NDPR52, and optineurin), and LC3 around parasitophorous vacuole membrane (PVM). STAT1-dependent upregulation of ISG15 stimulates deposition of adaptor proteins around PVM. This process is independent of Beclin 1 and does not cause vacuole lysosomal fusion. Instead, it results in entrapment of parasitophorous vacuole by multilayer membrane structure (*) and inhibition of parasite growth. **(B)** In endothelial cells infected with a type II strain of *T. gondii*, IFN-γ induces deposition of ubiquitin, p62, and NDPR52. This is followed by recruitment of Rab7, fusion with lysosomes, and parasite killing. Importantly, LC3 is not recruited around PVM, and this process is not dependent on key autophagy molecule ATG16L. Figure was generated using BioRender.com.

Although IFN-γ activates cell-autonomous control of *T. gondii* in mouse and human cells, there are significant differences in the effects of this cytokine between these two species. In this regard, IRGs and GBPs do not mediate anti-*T. gondii* activity in IFN-γ-stimulated human cells (Bekpen et al., 2005; Niedelman et al., 2013; Ohshima et al., 2014). Ubiquitination and some autophagy proteins are involved in the control of *T. gondii* induced by IFN-γ in human cells. However, these effector mechanisms are cell-type specific. In the case of human epithelial cells infected with type II or III (but not type I) strains of *T. gondii*, IFN-γ induces deposition of ubiquitin, p62, nuclear domain 10 protein 52 (NDP52), and LC3 around the PV (Selleck et al., 2015; Figure 5A). Although this process is dependent on ATG16L and ATG7, it does not represent autophagy, as it occurs independently of Beclin 1 and does not lead to VLF (Selleck et al., 2015). Although the integrity of the PV is maintained, a multilayer structure is formed around the PV that seems to explain the reduced growth rate of *T. gondii* (Selleck et al., 2015; Figure 5A).

IFN-γ also induces ubiquitination of the PV and recruitment of p62 and NDP52 in human endothelial cells (Clough et al., 2016; Figure 5B). However, in contrast to epithelial cells, LC3, GABARAP, and ATG16L are not recruited around the PV, and the induction of anti-*T. gondii* activity in endothelial cells is not dependent on ATG16L (Clough et al., 2016). Importantly, recruitment of ubiquitin and the adaptors p62 and NDP52 is followed by acidification of the PV and parasite killing (Clough et al., 2016; Figure 5B). This mechanism is operative against type II but not type I strains of *T. gondii* (Clough et al., 2016).

A recent study revealed that the presence of the ATG8 ortholog GABARAPL2 was key for the restriction of *T. gondii* replication in IFN-γ-activated HeLa cells (Zhang et al., 2020). GABARAPL2 accumulated around the PV in these cells in response to IFN-γ (Zhang et al., 2020). Similar to previous studies, IFN-γ induced deposition around the PV of ubiquitin and the adaptor proteins p62, NDP52, and optineurin (Zhang et al., 2020). These events are functionally relevant, as ubiquitination inhibitors or knockdown of the adaptor proteins impaired the recruitment of GABARAPL2 (Zhang et al., 2020). Moreover, knockdown of p62, NDP52, and optineurin impaired the ability of IFN-γ to reduce *T. gondii* replication. Finally, the recruitment of GABARAPL2 was dependent on the presence of ATG5, suggesting that lipidation of GABARAPL2 is required for its localization around the PV (Zhang et al., 2020). Thus, these studies, similar to those conducted in mice (Sasai et al., 2017), revealed the importance of GABARAPL2 in the control of *T. gondii* induced by IFN-γ.

Although IFN-γ sets in motion cell-autonomous immune responses to control *T. gondii* that is dependent on autophagy proteins, IFN-γ does not seem to upregulate ATG proteins. Recent studies were conducted to uncover the link between IFN-γ and the ATG pathway (Bhushan et al., 2020). IFN-γ upregulates IFN-stimulated genes (ISGs) (MacMicking, 2012). Work done in human epithelial cells revealed that ISG15 represents a molecular link between IFN-γ and the ATG pathway (Bhushan et al., 2020). ISG15 was markedly upregulated in IFN-γ-treated HeLa and A549 epithelial cells (Bhushan et al., 2020). Deletion of ISG15 partially inhibited the recruitment of p62, NDP52, and LC3 around the PV in IFN-γ-treated A549 cells without impairing ubiquitination of the vacuole (Bhushan et al., 2020; Figure 5A). The effect of ISG15 was selective, as the lack of this protein did not affect autophagy flux induced by rapamycin plus bafilomycin A1 (Bhushan et al., 2020). Moreover, ISG15 played an important role in the induction of IFN-γ-dependent restriction in *T. gondii* replication (Bhushan et al., 2020). Interestingly, ISG15^–/–^ mice did not have increased susceptibility to *T. gondii* (Napolitano et al., 2018). This may be explained by the lack of a known role for ISG15 in the recruitment of IRGs and GBPs, the major mechanism that mediates cell-autonomous control of *T. gondii* in mice.

## Potential Therapeutic Implications

Current antibiotic regimens against toxoplasmosis have significant adverse effects and are not proven to be effective in the case of ocular toxoplasmosis. Compounds that stimulate autophagy could become part of treatment regimens against *T. gondii*. Treatment of *T. gondii*-infected mammalian cells with rapamycin, a mTORC1 inhibitor and inducer of autophagy, triggered the killing of the parasite in a manner dependent on the presence of Beclin 1 (Andrade et al., 2006). Other studies evaluated the effects of docosahexaenoic acid (DHA), an omega-3 polyunsaturated fatty acid (Choi et al., 2019). DHA likely stimulated autophagy flux in mouse bone marrow-derived macrophages (BMDM) (Choi et al., 2019). In addition, DHA induced anti-*T. gondii* activity in BMDM that seemed to be mediated by autophagy, as DHA promoted the recruitment of LC3 around the PV, and 3-methyl adenine inhibited the anti-*T. gondii* activity induced by DHA (Choi et al., 2019). Similar findings were observed in BMDM from Fat-1 transgenic mice that synthesize omega-3 polyunsaturated fatty acid from ω6-PUFA (Choi et al., 2019). Moreover, Fat-1 mice infected with a type II strain of *T. gondii* exhibited lower tissue cyst counts in the brain (Choi et al., 2019).

More recent studies evaluated 4-hydroxybenzaldehyde (4-HBA) (Lee et al., 2020). 4-HBA-induced anti-*T. gondii* activity in BMDM seemed to be mediated by autophagy (Lee et al., 2020). 4-HBA seems to act *via* sirtuin-1 (SIRT1), a nicotine adenine dinucleotide-dependent protein deacetylase that can stimulate autophagy (Lee et al., 2020). 4-HBA upregulated SIRT1 protein levels, and SIRT1 inhibitors impaired the anti-*T. gondii* activity induced by 4-HBA. Although drugs that stimulate autophagy may facilitate the elimination of *T. gondii*, there are caveats with this approach. Autophagy regulates a broad range of homeostatic responses, including those in T cells, raising the concern that global stimulation of autophagy may have unintended negative consequences.

As part of an interesting approach, high-throughput screening of diverse small molecule libraries was performed in search of compounds that would enhance the anti-*T. gondii* activity of IFN-γ (Radke et al., 2018). A number of small molecules were identified that inhibited the growth of *T. gondii* at micromolar concentrations and exhibited increased potency in the presence of low concentrations of IFN-γ (Radke et al., 2018). Interestingly, some of these compounds increased the recruitment of LC3 around the PV, suggesting that they may act by potentiating the autophagy protein-mediated anti-*T. gondii* effects of IFN-γ (Radke et al., 2018). Compounds that synergize with immune mechanisms of control of *T. gondii* may improve the efficacy and tolerability of treatment regimens against toxoplasmosis.

The discovery that *T. gondii* manipulates host cell signaling to persistently block autophagic killing indicates that host-derived therapy may represent an attractive approach to improve the treatment of toxoplasmosis. Addition of Gefitinib, an EGFR TKI currently being used against cancer, to a broad range of human and rodent cells previously infected with *T. gondii* induced autophagic killing of type I and II strains of *T. gondii* (Lopez Corcino et al., 2019a). Importantly, Gefitinib was effective at concentrations up to ∼2 logs lower than those required for cytostatic activity against cancer cells (Lopez Corcino et al., 2019a). Gefitinib acted “on target” (required the presence of EGFR) and was active in the absence of immune activation of host cells. Moreover, *in vivo* administration of a relatively low dose of Gefitinib (∼1/4th to 1/12th of those used in cancer models) to mice with preestablished ocular and cerebral toxoplasmosis resulted in control of the disease (Lopez Corcino et al., 2019a). The protective effect of Gefitinib was not mediated by enhanced cellular or humoral immunity against *T. gondii* but was dependent on the normal expression of Beclin 1 (Lopez Corcino et al., 2019a). These findings, together with the demonstration that inhibition of EGFR or the upstream inducers of EGFR signaling results in autophagic killing of type I, II, or atypical strains of *T. gondii* in various human and mouse cells (Muniz-Feliciano et al., 2013; Portillo et al., 2017; Lopez Corcino et al., 2019a), identified EGFR as a therapeutic target against toxoplasmosis. A combination of low-dose EGFR TKI with antibiotics may lead to an improved regimen against toxoplasmosis.

## Conclusion

Maintaining the integrity of the PV is essential for the survival of *T. gondii*. CD40 stimulates autophagy that targets the PV resulting in the killing of *T. gondii*. Blocking the counterregulatory signaling induced by the parasite is sufficient to enable constitutive autophagy to target the PV and kill *T. gondii* (without the need for immune stimulation of autophagy). Studies to date indicate that in both scenarios, autophagic killing is not restricted to cell type, host species, or parasite strain. In contrast, IFN-γ triggers various autophagosome-independent mechanisms that attack the PV. The mechanisms activated by IFN-γ that have been identified thus far are not operative across species or parasite strains. Moreover, those uncovered in humans are not operative across cell types. Although much has been learned, further studies are needed to uncover the full spectrum of effector mechanisms induced by IFN-γ, especially in human cells, and identify the pathways of evasion utilized by *T. gondii*. This knowledge may contribute to the development of new approaches to improve the treatment of toxoplasmosis. The constitutive and conserved nature of autophagy already indicates that drugs that target components of the counterregulatory signaling pathway activated by *T. gondii* may become part of the novel and improved therapy against toxoplasmosis.

## Author Contributions

The author confirms being the sole contributor of this work and has approved it for publication.

## Conflict of Interest

The author declares that the research was conducted in the absence of any commercial or financial relationships that could be construed as a potential conflict of interest.
